# An Autonomous Vehicle Behavior Decision Method Based on Deep Reinforcement Learning with Hybrid State Space and Driving Risk

**DOI:** 10.3390/s25030774

**Published:** 2025-01-27

**Authors:** Xu Wang, Bo Qian, Junchao Zhuo, Weiqun Liu

**Affiliations:** School of Mechanical Engineering, Southwest Jiaotong University, Chengdu 610036, China; wangxu@catarc.ac.cn (X.W.); qianbo1588@my.swjtu.edu.cn (B.Q.); adolph@my.swjtu.edu.cn (J.Z.)

**Keywords:** autonomous vehicle, behavior decision, deep reinforcement learning, driving risk

## Abstract

Behavioral decision-making is an important part of the high-level intelligent driving system of intelligent vehicles, and efficient and safe behavioral decision-making plays an important role in the deployment of intelligent transportation system, which is a hot topic of current research. This paper proposes a deep reinforcement learning (DRL) method based on mixed-state space and driving risk for autonomous vehicle behavior decision-making, which enables autonomous vehicles to make behavioral decisions with minimal instantaneous risk through deep reinforcement learning training. Firstly, based on the various behaviors that may be taken by autonomous vehicles during high-speed driving, a calculation method for autonomous vehicle driving risk is proposed. Then, deep reinforcement learning methods are used to improve the safety and efficiency of behavioral decision-making from the interaction between the vehicle and the driving environment. Finally, the effectiveness of the proposed method is proved by training verification in different simulation scenarios, and the results show that the proposed method can enable autonomous vehicles to make safe and efficient behavior decisions in complex driving environments. Compared with advanced algorithms, the method proposed in this paper improves the driving distance of autonomous vehicle by 3.3%, the safety by 2.1%, and the calculation time by 43% in the experiment.

## 1. Introduction

Advanced Driver Assistance Systems (ADAS) play a critical role in modern intelligent transportation systems, enhancing both the intelligence and safety of vehicle operation [1,2,3]. Central to ADAS is the behavior decision-making function, which relies on the perception of various traffic participants within the driving environment [4,5,6]. With the rapid advancements in artificial intelligence, AI technologies have become integral to the assisted driving functions of autonomous vehicles, greatly enhancing the accuracy and reliability of these systems. In this context, the objective of this paper is to investigate the behavioral decision-making processes of autonomous vehicles through the application of deep reinforcement learning techniques.

When a vehicle operates on an open road, the dynamic changes in the state of surrounding traffic participants and the road environment, as captured in the scene information, can significantly influence the vehicle’s behavior. Consequently, lane change decisions must take into account the interactions within the surrounding driving context. Extensive research has been conducted on vehicle lane change decision-making, which can be broadly categorized into two primary approaches: rule-based decision-making methods and learning-based decision-making methods.

The rule-based decision-making approach assumes that a driver’s behavior is entirely rational, with lane change decisions based on factors such as safety, feasibility, obstacle locations, and the relative speed advantages of the current and target lanes [7]. The formulation of these rules is primarily based on considerations such as speed dissatisfaction, driving safety, and interactions with surrounding traffic participants [8,9,10]. Speed dissatisfaction is calculated by comparing the expected speed of the vehicle in front with the actual speed, which in turn influences the decision to change lanes [11]. Driving safety is concerned with the vehicle’s ability to avoid potential conflicts with surrounding entities, guiding decisions regarding lane changes or adjustments in speed (acceleration or deceleration) [12,13]. Interaction with surrounding traffic participants is typically modeled using game theory, which assumes a conflict of interest between the lane-changing vehicle and other surrounding vehicles. In this framework, decision-making rules for lane changes, acceleration, and deceleration are designed by optimizing for both safety and efficiency, thereby establishing a decision-making model for autonomous vehicle behavior [14,15,16].

On the one hand, the rule-based lane change decision-making approach may not fully capture the driving behaviors of human drivers. On the other hand, it can be challenging to implement, as it requires simultaneously considering and artificially representing the effects of multiple factors within a single calculation formula. In contrast, learning algorithms can autonomously learn vehicle driving parameters and represent more complex decision models, making them a key focus in the field of autonomous vehicle behavior decision-making. Currently, two primary types of learning-based decision-making methods are prevalent: deep learning and reinforcement learning.

The deep learning approach is characterized by its distributed processing and self-learning capabilities, which enable it to be trained using multiple sets of parameters that capture salient features. This training process leads to the accurate simulation of driving behavior and the optimization of model parameters, ensuring that the model’s output closely aligns with human behavior. For instance, Chen [17] developed a deep neural network (DNN) within the TORCS simulator environment, training vehicles to follow and overtake at high speeds. Xu [18] proposed a branch network structure of FCN-LSTM, integrating semantic segmentation techniques to better understand driving scene characteristics and predict vehicle movements in both horizontal and vertical directions probabilistically. Müller [19] introduced a vision-based convolutional neural network (CNN) for semantic segmentation of the road, enabling the prediction of driving strategies, including road identification and tracking via a PID controller. The training and testing for this model were conducted in the Carla simulator, albeit without traffic flow.

The effectiveness of reinforcement learning (RL) algorithms primarily hinges on the design of the reward function, as the manner in which rewards are assigned directly impacts model performance. Reward functions are typically categorized into two types: those that reward movement towards the goal and those that impose penalties for undesirable actions, such as collisions or deviations from the intended path. Kendall [20] demonstrated the application of RL in a real-world environment, where the vehicle was trained to follow lane markings. The training cycle ended when a safety officer determined that the vehicle had deviated from the road, with the reward function being defined as the maximum distance the vehicle traveled before being taken over. However, this approach, which uses a single scalar value as the reward, is limited in its ability to capture the complexity of real-world driving scenarios. A common improvement to reinforcement learning is its integration with deep learning techniques, forming what is known as deep reinforcement learning (DRL) [21,22,23,24,25]. One of the main advantages of reinforcement learning is that it does not require large amounts of human driving data; instead, it is trained based on maximizing the reward associated with specific behaviors. Moreover, since reinforcement learning is an online training process, it can simultaneously explore the environment and update the model. However, this approach suffers from relatively lower training efficiency [26]. To address this, Liang [27] proposed a method where a deep model is first trained using labeled data and then further optimized online using reinforcement learning strategies. This method shortens the training time compared to pure RL and leverages RL’s adaptability to improve model performance. Rhinehart [28] combined lidar data with imitation learning and model-based reinforcement learning to predict vehicle trajectories by mimicking expert behavior, with the training and testing conducted in the Carla simulator environment without dynamic traffic participants.

In contrast to the limitations of rule-based methods, such as restricted scene coverage, rigid procedural applications, and insufficient adaptability, learning-based decision-making approaches can leverage comprehensive vehicle driving scene information to acquire a broader range of experience [29]. Consequently, learning-based methods hold significant promise for wider applicability and offer greater flexibility in addressing dynamic driving environments. As the downstream of decision-making, planning can design specific driving trajectories based on the decision-making results, including lane change lateral planning and longitudinal speed planning [30,31].

This paper addresses the behavioral decision-making problem of autonomous vehicles on highways by employing a deep reinforcement learning approach, incorporating behavioral risk as a key factor. Behavioral risk enables autonomous vehicles to assess the level of danger associated with each decision, thereby providing a basis for the reward function in reinforcement learning. Deep neural networks are utilized to extract temporal features from driving scenarios represented by mixed state spaces. The reward function in this framework takes into account both driving risk and efficiency, guiding the autonomous vehicle to adopt safe and optimal behaviors. Initially, a method for calculating the driving risk of autonomous vehicles during high-speed driving is proposed, considering the various behaviors the vehicle may exhibit. Subsequently, deep reinforcement learning is applied to enhance both the safety and efficiency of the decision-making process, focusing on the interaction between the vehicle and the driving environment. Finally, the effectiveness of the proposed method is validated through training and evaluation across multiple simulation scenarios. Compared with advanced algorithm, the method proposed in this paper improves the driving distance of autonomous vehicle by 3.3%, the safety by 2.1%, and the calculation time by 43% in the experiment.

The structure of this paper is organized as follows: Section 2 outlines the methodology for calculating the risk associated with autonomous vehicle driving behaviors. Section 3 presents the design of a deep reinforcement learning approach for autonomous vehicle behavior decision-making. Section 4 involves training and simulating the proposed method, demonstrating its effectiveness and rationality. Finally, Section 5 concludes the paper with a summary of the findings and contributions.

## 2. Risk Analysis of Autonomous Vehicle Behavior

### 2.1. Behavior Model Construction of Autonomous Vehicle

In this paper, the quintic polynomial [32] is employed to model the lateral lane change behavior, while the Intelligent Driver Model (IDM) [33] is utilized to describe the longitudinal acceleration and deceleration dynamics of the vehicle.

The following assumptions are made for the quintic polynomial lateral lane change model: (1) the lateral position of the vehicle upon completion of the lane change is aligned with the center line of the target lane, (2) the vehicle’s longitudinal speed remains constant throughout the lane change, and (3) the lateral trajectory of the lane change is determined by a quintic polynomial. The lane change process governed by the quintic polynomial adheres to the following relationship.

(1) $$y=a_{0}+a_{1}t+a_{2}t^{2}+a_{3}t^{3}+a_{4}t^{4}+a_{5}t^{5}$$

where y represents the lateral offset during the lane change, and $a_{5},a_{4},a_{3},a_{2},a_{1},a_{0}$ are the coefficients of the quintic polynomial that define the trajectory of the lane change. The variable t denotes the time elapsed since the initiation of the lane change. From the lateral displacement function, lateral speed $v_{y}$ and lateral acceleration $a_{y}$ can be derived as the first and second derivatives, respectively. These quantities are essential for evaluating the dynamics of the lane change maneuver.

(2) $$\left\{\begin{array}{c} v_{y}=\dot{y}\left(t\right)=5a_{5}t^{4}+4a_{4}t^{3}+3a_{3}t^{2}+2a_{2}t+a_{1}\\ a_{y}=\ddot{y}\left(t\right)=20a_{5}t^{3}+12a_{4}t^{2}+6a_{3}t+2a_{2} \end{array}\right.$$

The specific shape of the polynomial trajectory is determined by the coefficient vector $A={\left[a_{0},a_{1},a_{2},a_{3},a_{4},a_{5}\right]}^{T}$. At the initial time $t=t_{0}$ of the lane change, the following boundary conditions are typically applied to ensure a smooth transition:

(3) $$Y\left(t_{0}\right)=Y_{0},v_{y}\left(t_{0}\right)=v_{0},a_{y}\left(t_{0}\right)=a_{0}$$

At the ending time $t=t_{end}$ of the lane change,

(4) $$Y\left(t_{end}\right)=Y_{end},v_{y}\left(t_{end}\right)=v_{end},a_{y}\left(t_{end}\right)=a_{end}$$

To determine the polynomial coefficients, six equations can be constructed based on the vehicle’s state at the start and end moments of the lane change:

(5) $$\left\{\begin{array}{l} a_{0}+a_{1}t_{0}+a_{2}{t_{0}}^{2}+a_{3}{t_{0}}^{3}+a_{4}{t_{0}}^{4}+a_{5}{t_{0}}^{5}=Y_{0}\\ a_{0}+a_{1}t_{end}+a_{2}{t_{end}}^{2}+a_{3}{t_{end}}^{3}+a_{4}{t_{end}}^{4}+a_{5}{t_{end}}^{5}=Y_{end}\\ a_{1}+2a_{2}t_{0}+3a_{3}{t_{0}}^{2}+4a_{4}{t_{0}}^{3}+a_{5}{t_{0}}^{4}=v_{0}\\ a_{1}+2a_{2}t_{end}+3a_{3}{t_{end}}^{2}+4a_{4}{t_{end}}^{3}+a_{5}{t_{end}}^{4}=v_{end}\\ 2a_{2}+6a_{3}t_{0}+12a_{4}{t_{0}}^{2}+20a_{5}{t_{0}}^{3}=a_{0}\\ 2a_{2}+6a_{3}t_{end}+12a_{4}{t_{end}}^{2}+20a_{5}{t_{end}}^{3}=a_{end} \end{array}\right.$$

Substituting the initial time $t_{0}=0$ and the end time $t_{end}=t_{end}$ into the polynomial will help simplify the system and determine coefficients:

(6) $$A=B^{-1}C$$

(7) $$A={\left[\begin{array}{ccc} a_{0} & a_{1} & \begin{array}{ccc} a_{2} & a_{3} & \begin{array}{cc} a_{4} & a_{5} \end{array} \end{array} \end{array}\right]}^{T}$$

(8) $$B=\left[\begin{array}{cccccc} 1 & 0 & 0 & 0 & 0 & 0\\ 0 & 1 & 0 & 0 & 0 & 0\\ 0 & 0 & 1 & 0 & 0 & 0\\ 1 & t_{\mathrm{end}} & {\left(t_{\mathrm{end}}\right)}^{2} & {\left(t_{\mathrm{end}}\right)}^{3} & {\left(t_{\mathrm{end}}\right)}^{4} & {\left(t_{\mathrm{end}}\right)}^{5}\\ 0 & 1 & 2t_{\mathrm{end}} & 3{\left(t_{\mathrm{end}}\right)}^{2} & 4{\left(t_{\mathrm{end}}\right)}^{3} & 5{\left(t_{\mathrm{end}}\right)}^{4}\\ 0 & 0 & 2 & 6t_{\mathrm{end}} & 12{\left(t_{\mathrm{end}}\right)}^{2} & 20{\left(t_{\mathrm{end}}\right)}^{3} \end{array}\right]$$

(9) $$C={\left[\begin{array}{ccc} Y_{0} & v_{0} & \begin{array}{ccc} a_{0} & Y_{end} & \begin{array}{cc} v_{end} & a_{end} \end{array} \end{array} \end{array}\right]}^{T}$$

In the context of lane change behavior, the state of the vehicle at the moment the lane change is initiated uniquely determines the entire lane change trajectory. Consequently, the quintic polynomial that describes the lane change is solely dependent on the lane change time.

The longitudinal IDM of the vehicle is represented as

(10) $$a\left(t\right)=\left\{\begin{array}{c} \max\left\{a_{max}\left[1-{\left(\frac{v\left(t\right)}{v_{0}}\right)}^{\delta }\right],a_{min}\right\},\;\mathrm{free}\;\mathrm{driving}\\ \max\left\{a_{max}\left[1-{\left(\frac{v\left(t\right)}{v_{0}}\right)}^{\delta }-{\left(\frac{s^{*}\left(t\right)}{s\left(t\right)}\right)}^{2}\right],a_{min}\right\},\;\mathrm{car}\;\mathrm{following} \end{array}\right.$$

(11) $$s^{*}\left(t\right)=s_{0}+\max\left(0,\;v\left(t\right)T-\frac{v\left(t\right)\dot{s}\left(t\right)}{2\sqrt{a_{max\;}a_{IDM}}}\right)$$

where $a_{max}=2\;m/s^{2}$ is the maximum acceleration that the vehicle can take, $a_{min}=-4\;m/s^{2}$ is the maximum deceleration that the vehicle can take, $v_{0}=30\;m/s$ is the expected speed, $v\left(t\right)$ is the speed of the vehicle at time t, $s\left(t\right)$ and $\dot{s}\left(t\right)$ are the relative distance and relative speed from the vehicle in front, $s_{0}=5\;m$ is the minimum distance between the two cars when they are stationary, T=1.5 s is the set distance between the front of the vehicle, which is the speed difference between the two vehicles at time t, and δ=4 and $a_{IDM}=4\;m/s^{2}$ is constant.

### 2.2. Classification Discussion and Risk Analysis of Driving Behavior

**Figure 1:**
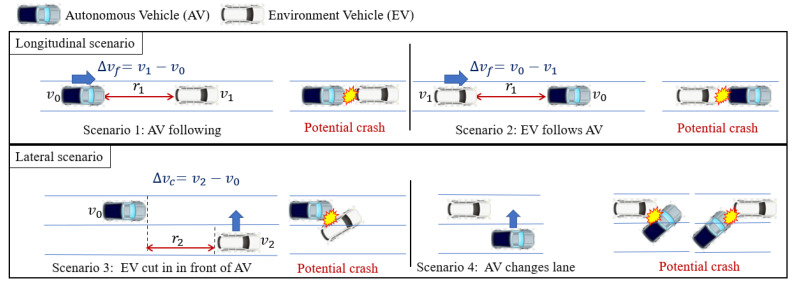
Potential collision under different behavior modes of an autonomous vehicle.

In scenarios where an autonomous vehicle interacts with an environmental vehicle on a structured road, two primary forms of behavior are observed: longitudinal follow (LF) and lateral lane change (LC). The former can be further categorized into three specific types, while the latter encompasses one distinct form. Potential collision risks associated with each of these behaviors are illustrated in Figure 1.

In Scenario 1, the autonomous vehicle is positioned in the same lane as the environmental vehicle and follows it, with the autonomous vehicle’s speed denoted as $v_{0}$, the environmental vehicle’s speed as $v_{1}$, the distance between them as $r_{1}$, and the relative speed as $∆v_{f}=v_{1}-v_{0}$. In Scenario 2, the autonomous vehicle is in the same lane but positioned ahead of the environmental vehicle, where the autonomous vehicle’s speed is $v_{0}$, the environmental vehicle’s speed is $v_{1}$, the distance between them is $r_{1}$, and the relative speed is $∆v_{f}=v_{0}-v_{1}$. In Scenario 3, the environmental vehicle is in an adjacent lane and is about to merge into the lane of the autonomous vehicle. The autonomous vehicle’s speed is $v_{0}$, the environmental vehicle’s speed is $v_{2}$, the longitudinal distance between them is $r_{2}$, and the relative speed is $∆v_{c}=v_{2}-v_{0}$. The acceleration of the autonomous vehicle in the longitudinal direction is represented by $a_{0}$, and the behavioral variable for the autonomous vehicle in Scenarios 1, 2, and 3 is $U=\left(a_{0}\right)$.

**Figure 2:**
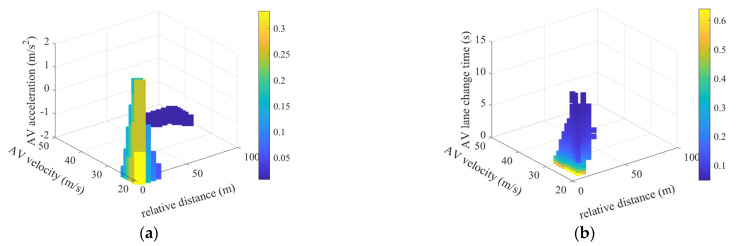
Risk of different actions of an autonomous vehicle in the case of lane following and lane change when the speed is 30 m/s: (**a**) action risk of car following; (**b**) action risk of lane change.

The longitudinal behavior risk of an autonomous vehicle is defined as follows. For a given scenario, the initial state S and the action of the environmental vehicle (with lane-following behavior characterized by constant speed and lane-change behavior defined by the lane change time t) together form a four-dimensional variable. The action $a_{0}$ of the autonomous vehicle is generated according to its behavior model. The actions of both the environmental vehicle and the autonomous vehicle are simulated, and if no collision occurs between the two vehicles, the action risk for the environmental vehicle in this scenario is considered zero. If a collision occurs, the action risk is calculated as the reciprocal of the two-vehicle collision time (TTC), which represents the risk of the environmental vehicle’s action (for lane-following, the action is acceleration, and for lane change, the action is lane change time). The shorter the collision time, the higher the risk associated with the action. Assuming a vehicle size of 5 m × 2 m for both the autonomous vehicle and the environmental vehicle, the four-dimensional variable combinations of lane-following and lane-changing actions can be traversed within their respective value ranges, enabling the calculation of the risk for various actions of the environmental vehicle in different scenarios. The longitudinal behavior risk for the autonomous vehicle traveling at a speed of 30 m/s is shown in Figure 2.

Scenario 4 represents a unique situation where the autonomous vehicle actively changes lanes. The primary focus of analyzing these four specific interaction forms is to assess the action risk of the vehicle, with the autonomous vehicle’s action being determined by its own intelligent driving algorithm. In this scenario, a classification-based approach is adopted to evaluate the lane change of the autonomous vehicle. Specifically, from the perspective of the environmental vehicle, the vehicle’s front is considered to be in the original lane before crossing the lane line. As the vehicle crosses the lane line, its front is considered to occupy both lanes until the rear of the vehicle completes the lane change, at which point the vehicle is considered to be entirely in the new lane.

Thus, two potential collision forms exist in Scenario 4: one involves a collision with the autonomous vehicle caused by a vehicle in the environment approaching from behind in the new lane during the lane change, and the other involves a collision with a vehicle in the environment in front of the new lane during the lane change. In these cases, the autonomous vehicle’s behavior corresponds to the conditions in Scenario 1 and Scenario 2, respectively, in the longitudinal direction. The associated risks can therefore be evaluated using the same longitudinal driving mode applied in these scenarios.

## 3. Design of Deep Reinforcement Model

### 3.1. Problem Description of Autonomous Vehicle Behavior Decision by DRL

Autonomous vehicle behavior decision-making can be modeled as a Markov Decision Process (MDP), which can be addressed using deep reinforcement learning algorithms. In this framework, the autonomous vehicle is considered the decision-making entity, known as the agent. The vehicle interacts with the driving environment, where its actions influence the state of both the agent and the surrounding environment. As the agent makes decisions, the environment responds, producing numerical rewards. The objective of the autonomous vehicle, as an agent, is to maximize the cumulative benefits derived from its actions.

In the MDP framework, the set of possible states of the agent and the environment is denoted as S, the set of actions that the agent can take is represented by A, and the rewards generated from those actions are denoted by R. At time t, the autonomous vehicle observes a specific state $s_{t}\in S$ of the environment and chooses an action $a_{t}\in A$. At the next time step t+1, the vehicle receives a reward $r_{t+1}\in R\subset R$ and the state of the environment is updated to $s_{t+1}$. Thus, the agent–environment interaction can be represented as a sequence of state–action–reward transitions:

(12) $$\left\{s_{0},a_{0},r_{1},s_{1},a_{1},r_{2},s_{2},a_{2},r_{3},\ldots ,s_{T},a_{T},r_{T+1}\right\}$$

where the subscript denotes the discrete time step, and T is the termination time. At each time step t, the state–action pair $\left(s_{t},a_{t}\right)$ leads to a new state and reward pair $\left(s_{t+1},r_{t}\right)$, with the corresponding probability distribution p∈γ that defines the dynamic characteristics of the MDP, i.e.,

(13) $$p\left(s_{t+1},r_{t}|s_{t},a_{t}\right)≝P\left\{S_{t}=s_{t+1},R_{t}=r_{t}|S_{t-1}=s_{t},A_{t-1}=a_{t}\right\}$$

The objective of MDP is to maximize the cumulative reward, represented by the expected value of the return $G_{t}$, i.e.,

(14) $$G_{t}≝R_{t+1}+\gamma R_{t+2}+\gamma^{2}R_{t+3}+\cdots =R_{t+1}+\gamma G_{t+1}=\sum_{k=0}^{\infty }\gamma^{k}R_{t+k+1}$$

where 0 ≤ γ ≤ 1 is the discount rate. The Markov Decision Process (MDP) framework aims to determine the optimal control policy by maximizing the expected cumulative rewards. The policy, denoted by π, defines the probability of selecting a specific action $a_{t}$ given a state $s_{t}$. The value function associated with a policy quantifies the expected return starting from a given state, under the guidance of the policy. Specifically, the state-value function $v_{\pi }\left(s_{t}\right)$ represents the expected cumulative reward from state s onward, following policy π. This function is mathematically expressed as

(15) $$v_{\pi }\left(s_{t}\right)=E_{\pi }\left(G_{t}|S=s_{t}\right)=E_{\pi }\left(\sum_{k=0}^{\infty }\gamma^{k}R_{t+k+1}|S=s_{t}\right)$$

Given the initial state–action pair $\left(s=s_{t},a=a_{t}\right)$, the action value function of the corresponding strategy π is the expectation of total return, which is denoted as $q_{\pi }\left(s_{t},a_{t}\right)$, quantifying how beneficial or detrimental the action is within the context of the policy, i.e.,

(16) $$q_{\pi }\left(s_{t},a_{t}\right)=\sum_{s^{\prime },r}p\left(s_{t+1},r_{t}|s_{t},a_{t}\right)\left[r_{t}+\gamma v_{\pi }\left(s_{t+1}\right)\right]$$

The optimal action-value function $q^{*}\left(s_{t},a_{t}\right)$ corresponds to the maximum expected return achievable from a given state–action pair, and it is defined as

(17) $$q^{*}\left(s_{t},a_{t}\right)=\arg\underset{\pi }{\max}q_{\pi }\left(s_{t},a_{t}\right)$$

If the optimal action value function $q^{*}$ can be obtained, the optimal action can be determined as

(18) $$a^{*}=\arg\underset{a}{\max}q^{*}\left(s,a\right)$$

By quantifying the driving behavior risk of autonomous vehicles, the numerical value τ of the behavior risk can be used as the return of MDP. Therefore, the optimal policy function for autonomous vehicle behavior decisions can be written as

(19) $$\pi^{*}\left(s\right)=\arg\underset{\pi }{\min}E_{\pi }\left(\sum_{i=0}^{\infty }\gamma^{i}\tau_{t+i}|s_{t}=s\right)$$

The equivalent is

(20) $$\pi^{*}\left(s\right)=\arg\underset{\pi }{\max}E_{\pi }\left(\sum_{i=0}^{\infty }\gamma^{i}\left(1-\tau_{t+i}\right)|s_{t}=s\right)$$

Therefore, the optimal action value function is written as

(21) $$q_{\pi }\left(s,a\right)=\arg\underset{\pi }{\max}E_{\pi }\left(\sum_{i=0}^{\infty }\gamma^{i}\left(1-\tau_{t+i}\right)|s_{t}=s,a_{t}=a\right)$$

The deep reinforcement learning method for solving the MDP process can be used to realize the trajectory planning of autonomous vehicles.

### 3.2. Deep Reinforcement Learning Method

**Figure 3:**
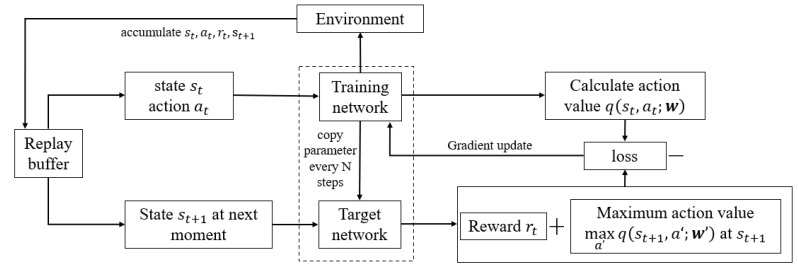
DQN framework.

To determine the optimal action value function $q^{*}$, a neural network with parameters w is utilized and denoted as $q\left(s,a;w\right)$. By training this neural network, an approximation of the optimal action value function can be achieved. The training process aims to minimize the loss, thereby obtaining an action value function with a high degree of approximation. This approach, known as Deep Q-Network (DQN), is implemented using Temporal Difference (TD) learning. The DQN framework consists of several key components, including the training network, target network, and experience replay pool. The experience replay mechanism stores the experiences generated by the agent through interactions with the environment. After selecting and executing an action using the ϵ-greedy strategy, the resulting reward and next state are stored as training samples. Both the training and target networks initially share the same set of parameters. The training network processes the current state and action from each sample to predict the Q-value of the selected action, while the target network processes the next state to predict the maximum Q-value over all possible actions. The training network adjusts its parameters based on each action, while the target network updates its parameters after a fixed time step, ensuring more stable training. The DQN framework has shown in Figure 3.

#### 3.2.1. Hybrid State Space

The state space consists of two parts: the autonomous vehicle state and the dynamic environment vehicle state.

The input status of the smart vehicle is represented as

(22) $${St}_{ego}=\left[\frac{x_{ego}-x_{0}}{x_{end}-x_{0}},\frac{y_{ego}}{\sum_{i=1}^{m}LW\left(m\right)}\right]$$

where $x_{ego}$ and $y_{ego}$ represent the longitudinal and lateral coordinates of the autonomous vehicle’s position, $x_{0}$ and $x_{end}$ denote the longitudinal coordinates of the starting and ending points of the road within the driving area, m is the number of lanes from the road’s starting point to the side of the driving direction of the autonomous vehicle, and LW represents the width of each lane.

The dynamic environment vehicle feature extraction takes into account the nearest vehicles positioned both in front and behind, as well as in adjacent lanes, within the autonomous vehicle’s perception range. A total of six vehicles are considered. The coordinates and speeds of these vehicles (with non-existent vehicles represented by a zero vector) are then converted into relative values with respect to the autonomous vehicle. This processed information forms the input state for the dynamic environment vehicle model, denoted as

(23) $${St}_{n}=\left[\frac{x_{n}-x_{ego}}{r_{d}},\frac{y_{n}-y_{ego}}{2LW}\right]$$

where the subscript n represents the index of the environmental vehicle. If no environmental vehicle is present at a specific location, its corresponding input state is set to a zero vector.

**Figure 4:**
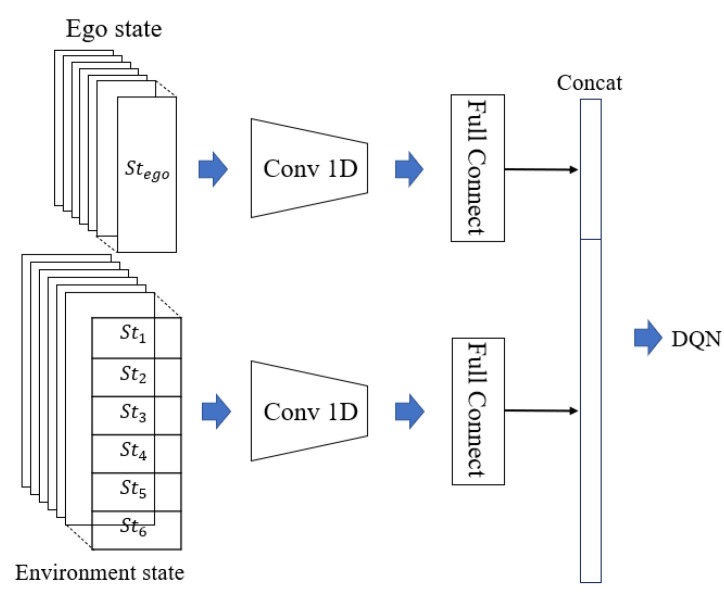
Feature extraction network backbone.

The state sequence of the autonomous vehicle’s state features and dynamic environmental entity features is extracted using a 1D convolutional layer, as shown in Figure 4. The feature extraction network backbone is then constructed through fully connected layers, with the extracted features concatenated and input as the state input for the DQN training network.

#### 3.2.2. Action Space

Action space A is designed for left-lane change, right-lane change, and longitudinal driving acceleration.

(24) $$A=\left\{\left\{t_{L}\right\},\left\{a\right\},\left\{t_{R}\right\}\right\}$$

where the set $\left\{t_{L}\right\}$ and $\left\{t_{R}\right\}$ contains the optional left lane change time, its value ranges from 3 s to 9 s, with intervals of 0.2 s. The set $\left\{a\right\}$ contains the optional longitudinal acceleration; its value ranges from −2 m/s^2^ to 2 m/s^2^, with intervals of 0.1 m/s^2^.

#### 3.2.3. Reward Function Design

The objective of this paper is to identify the driving behavior that minimizes vehicle risk, necessitating the incorporation of risk assessment results into the reward function of the DRL approach. To achieve this, a weighted combination of vehicle behavioral risk, driving efficiency, and driving comfort rewards is employed. These three types of rewards can be expressed as follows:

(25) $$\left\{\begin{array}{c} r_{r}=\sum_{t=1}^{t=T}\left(1-\tau_{t}\right)\\ r_{e}=\frac{1}{T}\sum_{t=1}^{t=T}\left(v_{t}-v^{*}\right)\\ r_{c}=-\left|a_{t+1}-a_{t}\right| \end{array}\right.$$

where $r_{r}$ is the behavior risk reward, $r_{e}$ is the vehicle driving efficiency reward, $r_{c}$ is the driving comfort reward, $\tau_{t}$ is the vehicle behavior risk value at time t, T is the total time for the vehicle to complete the driving task, $v_{t}$ is the longitudinal speed of the vehicle at time t, $v^{*}$ is the set cruising speed, and $a_{t}$ is the longitudinal acceleration at time t. The weighting of the three is

(26) $$r_{a}=\omega_{r}r_{r}+\omega_{e}r_{e}+\omega_{c}r_{c}$$

where $\omega_{r}$, $\omega_{e}$, and $\omega_{c}$ correspond to the weighting coefficients of the three rewards, respectively. Additionally, the total reward function incorporates the vehicle’s progress in completing the desired trajectory during the training process. Specifically, if the vehicle successfully completes the specified distance without incident, a completion reward is awarded; otherwise, a penalty for non-completion is imposed. Reward function is set as

(27) $$r=\left\{\begin{array}{c} 1+r_{a},complete\\ -20,\;otherwise \end{array}\right.$$

#### 3.2.4. Implement Step and Training Parameter

The steps of using the DQN algorithm to train vehicle behavior decisions are described in Algorithm 1.
**Algorithm 1.** DQN implementation processInput: Replay buffer size D, network update interval N, discount factor γ, learning rate α, reward function, state space, action space. Output: Parameters of training network and target network.
Initialize training network parameter w and target network parameter $w^{\prime }=w$For *i* = 1, D doa)Obtain environment vehicle state information as input of state space $s_{t}$b)For *t* = 1, *T* doPick random $a_{t}$ at small probability, otherwise $a_{t}=\arg\underset{a}{\max}q\left(s_{t},a;w\right)$Perform behaviour $a_{t}$, get reward $r_{t}$ and new environment state $s_{t+1}$Store $\left[s_{t},a_{t},r_{t},s_{t+1}\right]$ in replay bufferRandomly select batch $\left[s_{j},a_{j},r_{j},s_{j+1}\right]$ in replay buffer, calculate TD target $y_{j}=r_{j}+\gamma \;\underset{a}{\max}q\left(s_{j+1},a;w^{\prime }\right)$Use gradient descent to update parameter $w=w-\alpha \left(q_{j}-y_{j}\right)\frac{\partial q\left(s_{j},a_{j};w\right)}{\partial w}$Copy network parameter w to $w^{\prime }$ every N stepc)End forEnd for


Each iteration in the algorithm updates the neural network parameters to learn the optimal action value, thus enabling the approximation of the action value function. Table 1 outlines the hyperparameter settings used during the algorithm’s training process.

## 4. Experiment and Discussion

**Figure 5:**
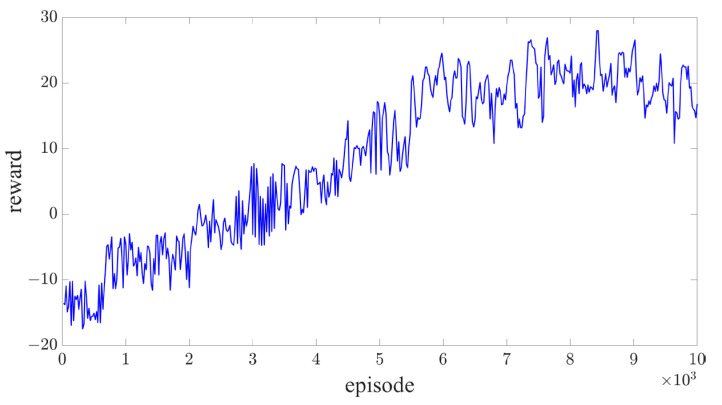
Training process reward curve.

The intelligent driving simulation platform CARLA provides standardized road networks, various vehicle control models, and precise sensor data, making it an ideal environment provider for the deep reinforcement learning framework employed in this paper. The proposed autonomous vehicle behavior decision-making algorithm is trained within the CARLA simulator. The environmental vehicle control model utilizes the built-in algorithm of the simulator, and the initial positions of both the autonomous vehicle and surrounding traffic participants are randomly assigned within a predefined area for each training batch. The vehicle begins from its starting position, and the end of the driving process is determined either by a collision or after traveling a distance of 200 m. The reward during the training process is recorded as Figure 5, and analysis of the reward curve indicates that the proposed algorithm converges rapidly.

**Figure 6:**
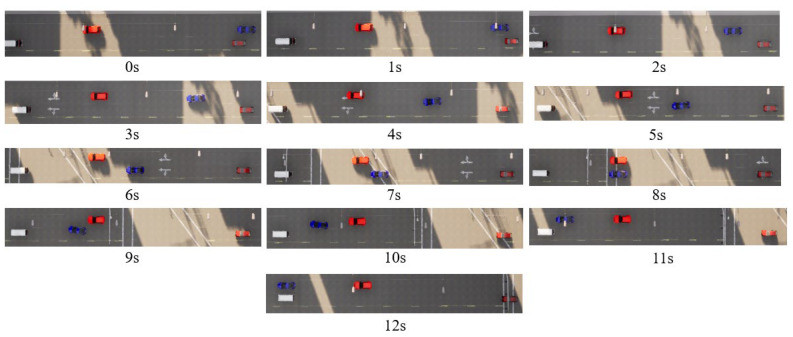
Bird’s-eye view of two-lane scenario road test (typical case for scenario 1).

**Figure 7:**
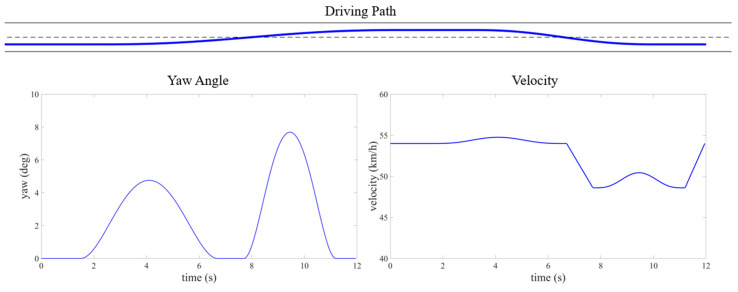
Kinematic curve of two-lane scenario road test (typical case for scenario 1).

**Figure 8:**
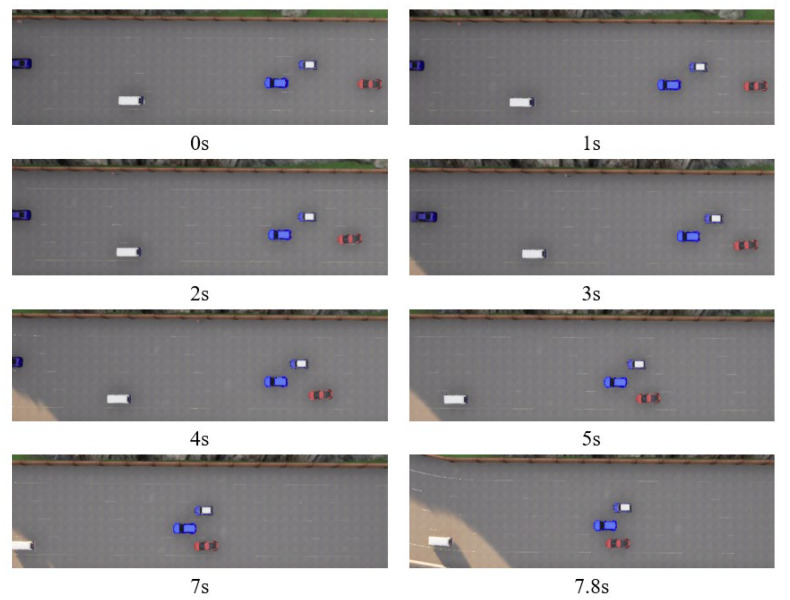
Bird’s-eye view of four-lane scenario road test (typical case for scenario 2).

**Figure 9:**
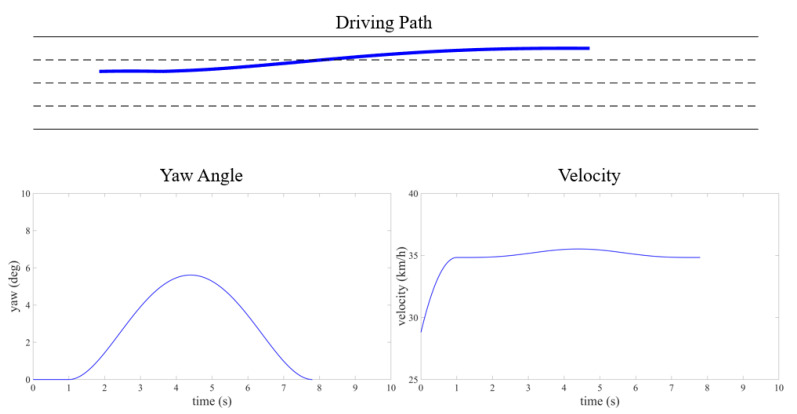
Kinematic curve of four-lane scenario road test (typical case for scenario 2).

The training results are evaluated across various traffic scenarios, which include different road configurations and environmental vehicle driving behaviors. The autonomous vehicle successfully completes driving tasks in these diverse driving conditions. In Scenario 1, the road is a one-way, two-lane layout, where three randomly generated environmental vehicles are all traveling at a constant speed of 10 m/s, with no vehicles driving side by side. The autonomous vehicle starts at the center of the right lane, positioned behind one of the environmental vehicles, with an initial speed of 15 m/s. Its target cruising speed is also set to 15 m/s. In Scenario 2, the road is a one-way, four-lane configuration, with multiple randomly generated environmental vehicles driven using CARLA’s built-in autonomous driving model to create a traffic flow. The method of generating the position and velocity of the environmental vehicle is statistically significant. The initial velocities are randomly selected between 10 and 20 m/s, the initial longitude positions are randomly selected within 50 m in front of the autonomous vehicle, and the lateral position is selected at the center of random lane. The autonomous vehicle starts at the centerline of a random lane at the rear of the generated traffic flow. Some typical cases from both scenarios are shown in Figure 6, Figure 7, Figure 8 and Figure 9.

In the two experimental scenarios, the results demonstrate that the behavior decision-making method based on driving risk, as proposed in this paper, allows autonomous vehicles to make effective decisions in complex driving environments. To evaluate the impact of the behavior risk value on improving the behavior strategy of autonomous vehicles, a comparative experiment was conducted, with the results presented in Table 2. The comparison includes the baseline method (where vehicle behavior is entirely randomly generated), numerical-optimization-based EM-Planner method, deep-learning-based LSTM method, DRL-based DQN method with the behavior risk input removed, and DRL-based DDPG method. The evaluation metrics for the behavior decision-making algorithms are the average driving distance x, the standard deviation of the driving distance σ, the number of collisions with other vehicles or road edges NoC, and average computation time ACT (unit: ms), all computed over multiple random experimental scenarios.

The comparison among baseline and other methods highlights that the application of numerical optimization and learning-based methods significantly enhance the safety and precision of autonomous vehicle behavior decision-making. The numerical optimization method can show excellent reliability, but its disadvantage is that the search for the feasible solution space consumes a huge amount of computational time, so the real-time performance is unsatisfactory. Compared with reinforcement learning methods, deep learning methods rely too much on the input dataset, and the computation time is strongly correlated with the complexity of network design, so its generalization is poor. The advantages of the DRL method based on driving risk are evident in the comparison. Firstly, in terms of computational time, the DRL method is significantly better than the numerical optimization method and deep learning method. Secondly, through the comparison of the DQN method without introducing vehicle behavior risk and the DDPG method with more complex network structure to the proposed method, it is evident that incorporating behavior risk further improves decision-making performance. The integration of behavior risk allows the autonomous vehicle to assess potentially hazardous actions that could lead to collisions at each decision point, thus promoting safer behavior patterns across various driving conditions. This, in turn, enhances the overall safety of the autonomous vehicle. While the reward function in traditional DRL methods primarily penalizes collisions with a large negative reward, the DRL method based on vehicle behavior risk, as presented in this paper, applies negative rewards to dangerous actions from the outset of each decision-making cycle. This results in improved overall driving performance throughout the vehicle’s driving process. Compared with the current advanced DDPG algorithm, the DQN method with driving risk proposed in this paper improves the driving distance of autonomous vehicle by 3.3%, the safety by 2.1%, and the calculation time by 43% in the experiment.

## 5. Conclusions

This paper presents a deep reinforcement learning method based on a mixed state space and driving risk for autonomous vehicle behavior decision-making. The goal is to enable autonomous vehicles to make decisions that minimize instantaneous risk based on real-time traffic conditions. The proposed numerical calculation method for behavior risk allows the autonomous vehicle to assess the danger level of its actions at each decision-making point, which is integrated into the reward function of the DRL model. Experimental results demonstrate that the proposed DRL approach effectively guides autonomous vehicles to adopt both efficient and safe driving behaviors. The key advantages of the proposed method are as follows: (1) the reward function accounts for both driving risk and efficiency, (2) the autonomous vehicle gains awareness of potentially dangerous behaviors that may lead to collisions, allowing it to adopt safer driving strategies under varying conditions, and (3) the DRL approach directly applies negative rewards to dangerous actions at each decision point, thereby improving the overall driving performance of the autonomous vehicle. Future work will focus on the comparison of different methods’ effect on efficiency, occupied computing resources, and practical performance in real traffic environment.

## Figures and Tables

**Table 1:** Algorithm hyperparameter setting.

Hyperparameter	Symbol	Value
discount factor	γ	0.99
learning rate	α	0.001
replay buffer size	D	5000
network update interval	N	5
batch size		128
weighting coefficients of risk	$\omega_{r}$	0.5
weighting coefficients of efficiency	$\omega_{e}$	1
weighting coefficients of comfort	$\omega_{c}$	0.1

**Table 2:** Performance evaluation indicators of different behavioral decision-making methods.

Indicator	Scenario 1—Two Lanes				Scenario 2—Four Lanes			
	x (m)	σ (m)	NoC	ACT (ms)	x (m)	σ (m)	NoC	ACT (ms)
Baseline	15.3	8.2	100	-	19.5	9.2	100	-
EM-Planner [34]	170.7	4.2	5	109.3	206.9	8.5	7	124.7
LSTM [35]	142.8	11.2	18	52.9	168.4	11.4	16	53.5
DQN-no risk	62.5	14.7	57	2.84	73.2	11.8	44	2.88
DDPG [36]	174.8	3.9	4	4.96	210.5	8.1	5	5.13
DQN (this paper)	179.5	3.3	2	2.85	218.7	7.8	3	2.93

## Data Availability

Data available on request from the authors.
